# Increase of Oxidative Stress by Deficiency of The ALDH2/UCP2/Nrf2 Axis Exacerbates Cardiac Dysfunction in Chronic Kidney Disease 

**DOI:** 10.31083/j.rcm2304127

**Published:** 2022-04-02

**Authors:** Lei Xu, Shasha Han, Zhaoyang Chen, Cheng Shen, Zihan Yao, Peng Wang, Yunzeng Zou, Aijun Sun, Junbo Ge

**Affiliations:** ^1^Department of Cardiology, Zhongshan Hospital, Fudan University, Shanghai Institute of Cardiovascular Diseases, 200032 Shanghai, China; ^2^Intensive Care Unit, Jining First People’s Hospital, 272002 Jining, Shandong, China; ^3^Heart Center of Fujian Province, Union Hospital, Fujian Medical University, 350401 Fuzhou, Fujian, China; ^4^Department of Cardiology, Affiliated Hospital of Jining Medical University, Jining Key Laboratory for Diagnosis and Treatment of Cardiovascular Diseases, 272007 Jining, Shandong, China; ^5^Department of Clinical Pharmacy, People's Hospital of Putuo District, 200060 Shanghai, China; ^6^Health Service Center of Yadong County, Shigatse City, 857600 Tibetan Autonomous Region, China; ^7^Institute of Biomedical Science, Fudan University, 200032 Shanghai, China

**Keywords:** aldehyde dehydrogenase 2 (ALDH2), nephrectomy, cardiac hypertrophy, reactive oxygen species (ROS)

## Abstract

**Background:**

Both epidemiologic and experimental studies have evidenced that chronic kidney disease (CKD) could increase the incidence and risk of cardiac dysfunction, especially in aging patients. However, the underlying mechanisms are still not fully understood.

**Methods:**

In this study, we used 8 weeks old male wild-type (WT) C57BL/6 mice and ALDH2 knockout (ALDH2-/-) mice with C57BL/6 background. Here the 5/6 nephrectomy (NX) mouse model was constructed to study how CKD affects cardiac function and explored the related role of aldehyde dehydrogenase 2 (ALDH2), a well-established cardioprotective factor, in this process.

**Results:**

Compensatory cardiac hypertrophy was found in wild type (WT) mice 12 weeks post 5/6 NX as shown by increased left ventricular wall thickness (LVWD), cross-sectional area (CSA) of cardiomyocytes, and preserved left ventricular ejection fraction (EF) and fractional shorten (FS). Deficiency of ALDH2 (ALDH2-/-) significantly reduced EF and FS as compared with WT mice 12 weeks post 5/6 NX, while left ventricular hypertrophy was similar between the two NX groups. ALDH2-/- CKD groups showed more severe nephritic damages and increased fibrosis deposition in hearts. Besides, levels of reactive oxygen species (ROS) and apoptosis were also significantly upregulated in hearts of ALDH2-/- NX mice. The above changes were related with decreased expressions of uncoupling protein 2 (UCP2) and nuclear factor like 2 (Nrf2), as well as the downstream effectors of Nrf2 (heme oxygenase-1, HO-1 and superoxide dismutase 2, SOD2).

**Conclusions:**

Our data indicated that ALDH2 deficiency did not affect NX-induced left ventricular hypertrophy, but could increase oxidative stress and exacerbate CKD-induced cardiac dysfunction, partly via downregulation of UCP2 and Nrf2/ARE (antioxidant response element) pathways.

## 1. Introduction 

The importance of global aging is significant for the social and health systems. Elderly people have an increased prevalence of chronic kidney disease (CKD), which is related to higher risk of hospitalization, death, and costs [1]. There is also a link between CKD and increased incidence of cardiovascular diseases (CVDs), which is the leading cause of death in CKD patients [2]. The presence of CKD could result in left ventricular hypertrophy, arrhythmia, and ultimately heart failure [3], with left ventricular hypertrophy being the most common and most typical cardiac change in CKD patients [4]. It is known that the initial hypertrophic response induced by CKD is an important compensatory process and thought to be favorable for maintaining the normal heart function [5]. Nonetheless, prolonged CKD might eventually induce pathological cardiac remodeling and heart failure [3]. Multiple pathophysiologic mechanisms contribute to the development of cardiac damage in CKD patients, including inflammation, hypertension, abnormal blood lipids, and increased volume load [6]. Recently, studies have also indicated that the imbalance of oxidation and antioxidation is responsible for the cardiac dysfunction induced by CKD [7]. However, the precise pathological mechanisms have remained unclear.

Aldehyde dehydrogenase 2 (ALDH2) is an essential mitochondrial enzyme that plays a key role in ethanol metabolism. Accumulating results from our team and others indicate that ALDH2 also plays a protective role in ischemic, toxic, and pressure overload-induced cardiac damages [8, 9, 10], as well as attenuating myocardial remodeling and age-related contractile dysfunction [11]. Apart from metabolizing acetaldehyde into innocuous acetic acid, ALDH2 also participates in eliminating superoxide and reactive stress, which subsequently modulates apoptosis, necrosis, and autophagy in cardiomyocytes [12, 13]. Xu *et al*. [14] reported that activation/overexpression of ALDH2 were associated with increased and decreased renal injury in murine models of acute kidney injury. Nonetheless, it remains unclear whether ALDH2 also participates in the pathogenesis of CKD-induced cardiac dysfunction. The imbalance of reactive stress derives from discorded oxidative stress and decreased expressions of antioxidative proteins. Uncoupling protein 2 (UCP2), which is located in the mitochondrial inner membrane, has been found to protect the cardiovascular system by supressing the generation of reactive oxygen species (ROS) and oxidative stress [15, 16]. Nuclear factor like 2 (Nrf2) is a transcription factor and is recognized as a known antioxidant. It can translocate to the nuclear area and bind with antioxidant response elements of the promoter regions of encoding genes such as heme oxygenase-1 (HO-1) and superoxide dismutase 2 (SOD2), protecting ROS generation and excessive reactive stress in the myocardia [17, 18]. Herein, we tested the hypothesis that ALDH2 deficiency might exacerbate CKD-induced cardiac dysfunction by increasing ROS generation, and explored related molecular mechanisms.

## 2. Methods 

### 2.1 Animals 

All experimental procedures involving animals in this study were approved by the Animal Care Committee of Zhongshan Hospital, Fudan University. In this study, we used 8 weeks old male wild-type (WT) C57BL/6 mice and ALDH2 knockout (ALDH2-/-) mice with C57BL/6 background. The C57BL/6 mice were purchased from Shanghai Jiesijie Laboratory Animal Centre (Shanghai, China), and ALDH2-/- mice were made as our previous article [13]. All mice were in a room with light- and temperature-controlled situation and normal diet. After one week of environmental acclimatization, mice were randomly assigned into chronic kidney disease groups (CKD) and sham-operated groups (Sham). The CKD groups were induced by a 2-step 5/6 nephrectomy (NX) as described previously to establish a CKD model [19]. The Sham group was treated in the same way as described for CKD without manipulation of the kidneys. The total sample size was 28, including 7 mice in each groups. Though all groups were used intraoperative analgesics and intra- and post-operative and antibiotics to reduce the mortality and the potential infections, 4 mice died (2 in ALDH2-/- CKD group, 1 in WT CKD group and 1 in ALDH2-/- Sham group).

### 2.2 Echocardiographic Assessment 

At 12 weeks after 5/6 NX, echocardiography was carried out with a VisualSonics ultrasound imaging system (VisualSonics Vevo770, VisualSonics Inc., Toronto, Canada) equipped with a linear 30 MHz high-frequency probe as our previous article [13]. All mice were anesthetized with 3% isoflurane inhalation, and then maintained with 1% isoflurane pending the echocardiographic detection. After obtaining 2-dimensional left ventricular long-axis images, M-mode traces were adjusted for the acquisition t of left ventricular end-diastolic dimension (LVEDD), left ventricular end systolic dimension (LVESD), left ventricular diastolic anterior wall thickness (LVAWD), left ventricular diastolic posterior wall thickness (LVPWD), ejection fraction (EF), and fractional shortening (FS). All measurements were averaged for at least 5 consecutive cardiac cycles. Inter- and intra- observer variability was assessed by calculating the differences between the values of 10 randomly selected subjects measured by one observer twice and by a second observer.

### 2.3 Histological Analysis 

Mice were sacrificed after echocardiographic examination under deep anesthesia (pentobarbital sodium, 150 mg/kg, i.p.), followed by removal of the heart, lung, and kidney. Heart tissues and kidney tissues assigned for histological analysis were then placed in 4% neutral formalin at room temperature for more than 24 h and were embedded in paraffin. The cables were cut into 5 μm thickness and stained with hematoxylin and eosin (H&E) and Sirius red as previously described [20]. At least 5 random high-resolution fields of cross-sectional area of cardiomyocytes (400×) or red stained collagen fibers (200×) of each mouse were captured and examined by Image-J software (National Institutes of Health, Bethesda, MD, USA).

### 2.4 Intracellular Fluorescence Measurements of ROS and Apoptosis 

Frozen sections of the heart were treated as previously described and were used to evaluate the levels of ROS and apoptosis of cardiomyocytes with or without 5/6 NX surgeries [13]. The intracellular ROS and apoptosis were determined by the Fluorometric intracellular ROS kit (Sigma-Aldrich, St. Louis, MO, USA) and Fluorescein In Situ Cell Death Detection Kit (Roche Diagnostics, Basel, Switzerland), respectively. Photos were taken by high-resolution digital image analysis system (QwinV3, Leica, Wetzlar, Germany) at magnification of 200× or 400×. A total of 5 random high-power fields from each heart sample were chosen and quantified in a blinded manner.

### 2.5 Western Blot Analysis 

Equal amounts of protein from each sample were resolved on sodium dodecyl sulfate polyacrylamide gels (SDS-PAGE, Bio-Rad, Hercules, CA, USA), and then transferred to polyvinylidene fluoride (PVDF) membranes (Millipore, Burlington, MA, USA). After blocking by Tris-buffered saline (TBS) buffer containing 5% bovine serum albumin (BSA), the membranes were incubated with primary antibodies (anti-ALDH2 (1:3000, Novus Biologicals, Littleton, CA, USA), anti-UCP2 (1:1000, Cell Signaling Technology, Danvers, MA, USA), anti-Nrf2 (1:1000, Cell Signaling Technology), anti-SOD2 (1:1000, Cell Signaling Technology), anti-HO-1 (1:1000, Cell Signaling Technology) overnight at 4 ℃ in TBST buffer. The membranes were then incubated with secondary antibodies at 1:4000 for 90 min at room temperature. We used an enhanced chemiluminescence detection technique for imaging and the expressions of proteins were measured and analyzed by Quantity One software (1709605, Bio-Rad, USA).

### 2.6 Statistical Analysis 

All data were expressed as mean ± standard error of the mean (SEM). Analyses were performed with statistical software SPSS 16.0 (IBM Corp., Chicago, IL, USA). The differences among 4 groups were analyzed by one-way analysis of variance (ANOVA) followed by least significant difference (LSD) analysis. *p *< 0.05 was considered statistically significant.

## 3. Results 

### 3.1 ALDH2 Deficiency Exacerbates CKD-induced Cardiac Dysfunction 

**Fig. 1.**
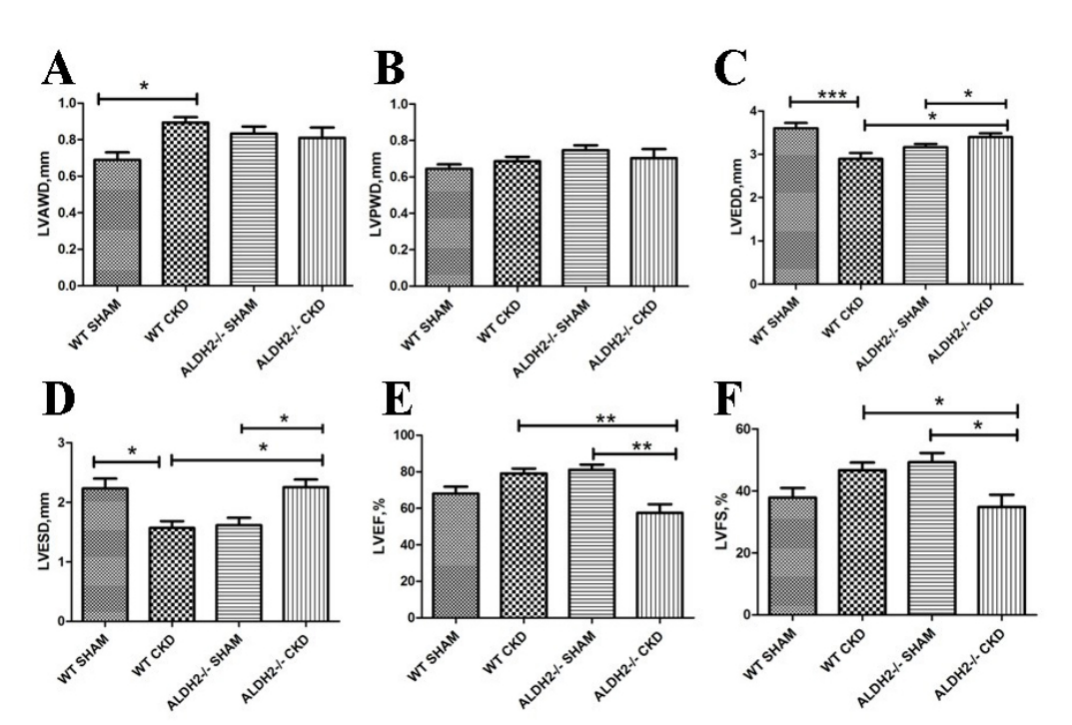
**Cardiac function of WT and ALDH2-/- mice post 5/6 NX**. (A) LVAWD. (B) LVPWD. (C) LVEDD. (D) LVESD. (E) LVEF. (F) LVFS. Values are presented as mean ± SEM (n = 5–7 per group). *p < 0.05, **p < 0.01, ***p < 0.001. WT, wild type; ALDH2, aldehyde dehydrogenase 2; LVAWD, left ventricular diastole anterior wall thickness; LVPWD, left ventricular diastole posterior wall thickness; LVEDD, left ventricular end-diastolic dimension; LVESD, left ventricular end-systolic dimension; LVEF, left ventricular ejection fraction; LVFS, left ventricular fractional shortening.

**Fig. 2.**
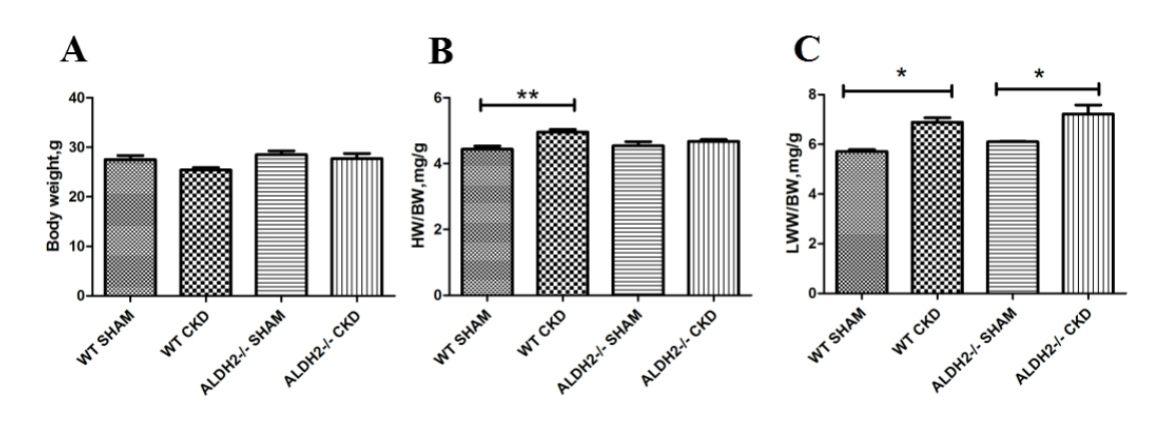
**General features of WT and ALDH2-/- groups post 5/6 NX**. (A) Body weight of each group. (B) HW/BW. (C) LWW/BW. Values are presented as mean ± SEM (n = 5–7 per group). * *p *< 0.05, ** *p *< 0.01. WT, wild type; ALDH2, aldehyde dehydrogenase 2; HW/BW, heart weight/body weight; LWW/BW, lung wet weight/body weight.

As shown in Fig. 1, the LVAWD increased significantly in WT with 5/6 NX mice. Meanwhile, the LVEDD and LVESD reduced in the WT with CKD group. However, the LVEDD and LVESD increased in ALDH2-/- with CKD mice compared with both the ALDH2-/- Sham group and WT CKD group. Besides, although CKD challenge did not affect the EF and FS in WT mice, these parameters were significantly reduced in ALDH2-/- CKD mice. The comparisons of the echocardiographic parameters between WT Sham and ALDH2-/- Sham showed no statistically significant differences (LVAWD *p* = 0.125, LVEDD *p* = 0.069, LVESD *p* = 0.057, LVPWD *p* = 0.072, EF *p* = 0.173, FS *p* = 0.088). The above results indicated that CKD might induce the compensatory hypertrophic response in WT mice as evidenced by increased LVAWD and normal EF and FS values, while ALDH2 deficiency resulted in cardiac systolic dysfunction and aggravated left ventricle enlargement without hypertrophy. In Fig. 2, 5/6 NX exposures remarkably increased the heart weight/body weight (HW/BW) in WT mice instead of in ALDH2 deficiency groups, and the lung wet weight/body weight (LWW/BW) was greater in WT and ALDH2-/- groups with CKD than the corresponding sham groups. These results suggested that ALDH2 deficiency could exacerbate CKD-induced cardiac dysfunction, but did not affect left ventricular hypertrophy; left ventricular hypertrophy was observed only in WT with CKD group.

### 3.2 Histological Changes Post 5/6 NX Related to ALDH2 Deletion 

**Fig. 3.**
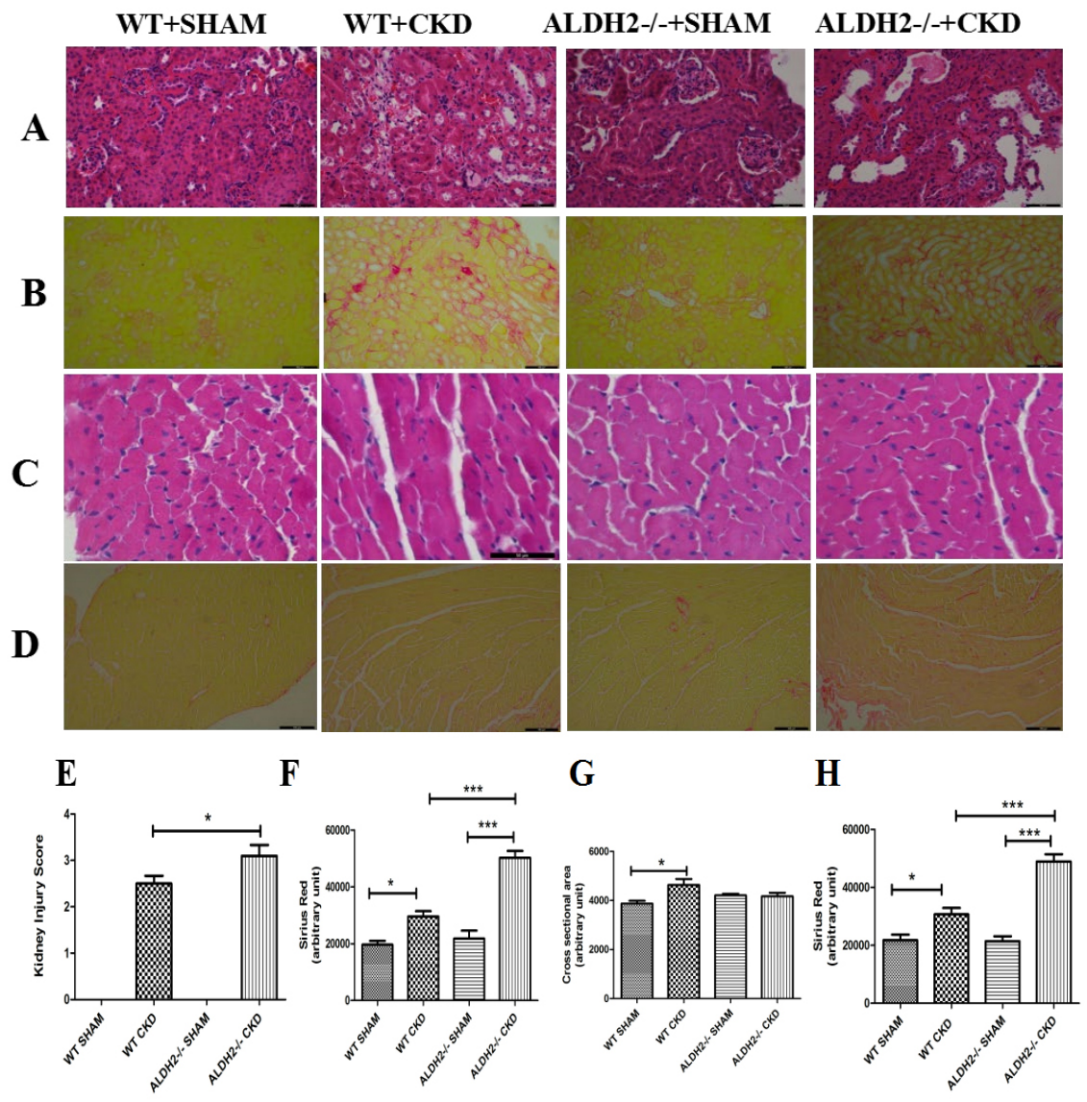
**Analysis of renal and myocardial cross-sectional areas and fibrosis in WT and ALDH2-/- groups post 5/6 NX**. (A) Representative images of H&E staining in kidney. (B) Representative images of Sirius Red staining in kidney. (C) Representative images of H&E staining in heart. (D) Representative images of Sirius red staining in heart. (E) Kidney injury score for each group. (F) Quantitative analysis of kidney fibrosis. (G) Quantitative analysis of myocardial cross-sectional areas. (H) Quantitative analysis of myocardial fibrosis. Values are expressed as mean ± SEM (n = 3–5 per group). * *p *< 0.05, *** *p *< 0.001. WT, wild type; ALDH2, aldehyde dehydrogenase 2.

To investigate the influence of ALDH2 on CKD-induced renal and myocardial histological changes, cross-sectional areas and fibrosis deposition were examined. The kidney injury score was calculated using semi-quantitative appraisal based on the kidney’s histopathological condition. The appraisals were given 0–4 scales as follows: 0, none; 1, tubular injury <10% of random areas; 2, tubular injury <25% of random areas; 3, tubular injury <75% of random areas; and 4, tubular injury >75% of random areas [21]. Analysis of the kidneys images of H&E and Sirius red staining (Fig. 3A,B) revealed that obvious damages were found post 12 weeks of 5/6 NX both in WT and ALDH2-/- mice, as evidenced by swollen glomeruli and increased fibrosis deposition in kidneys (Fig. 3E,F). Meanwhile, ALDH2 deficiency exacerbated the nephritic damages post 5/6 NX. As shown in Fig. 3C,D, cross-sectional areas in WT mice were significantly enlarged as compared with the ALDH2-/- CKD group. Besides, increased fibrosis deposition in hearts was only found in ALDH2-/- CKD mice as compared with the sham group (Fig. 3G,H).

**Fig. 4.**
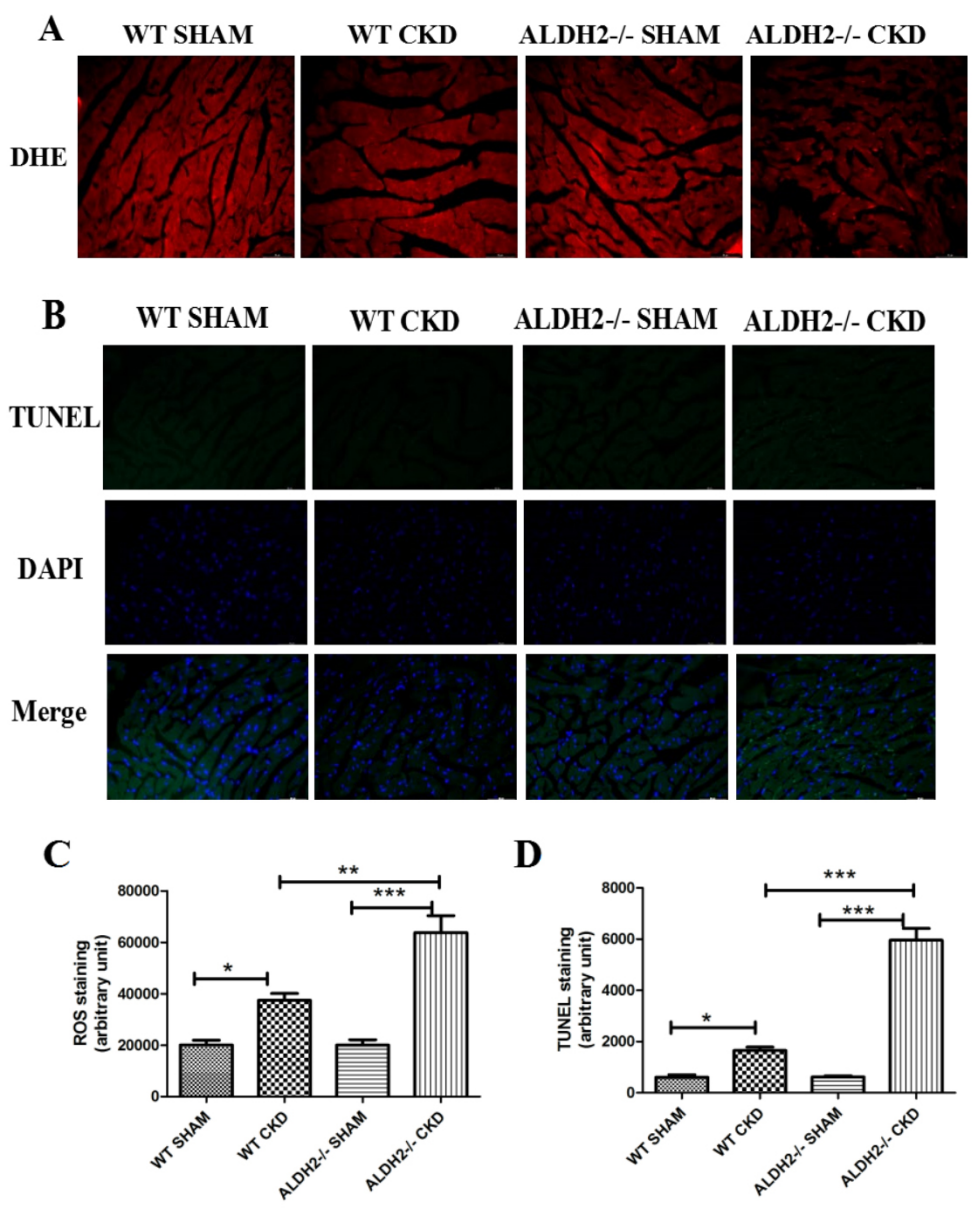
**Analysis of ROS and apoptosis in WT and ALDH2-/- mice post 5/6 NX**. (A) Representative Images of DHE staining. (B) Representative images of TUNEL staining. (C) Quantitative analysis of myocardial ROS levels. (D) Quantitative analysis of myocardial apoptosis. Values are presented as mean ± SEM (n = 3–5 per group). * *p *< 0.05, ** *p *< 0.01, *** *p *< 0.001. ROS, reactive oxygen species; WT, wild type; ALDH2, aldehyde dehydrogenase 2.

Previous findings have shown that ALDH2 plays a crucial role in detoxification of reactive aldehydes and suppressing oxidative stress [12]. We further assessed whether this pathological stress could participate in the reduced cardiac function in ALDH2-/- mice post CKD challenge. As shown in Fig. 4A, 5/6 NX itself could increase ROS accumulation in WT andCKD mice. Furthermore, it was remarkably increased in ALDH2/- CKD group as compared with the WT CKD group. Considering that redundant ROS could trigger mitochondrial apoptosis, apoptosis of cardiomyocytes was determined by terminal deoxynucleotidyl transferase dUPT nick end labeling (TUNEL) assay (Fig. 4B). Consistent to the changes of ROS, the number of apoptotic cells was more in ALDH2-/- group than the WT post 5/6 NX group (Fig. 4C,D).

### 3.3 Impact of ALDH2 Deficiency on The Expression of Anti‑oxidative Proteins 

**Fig. 5.**
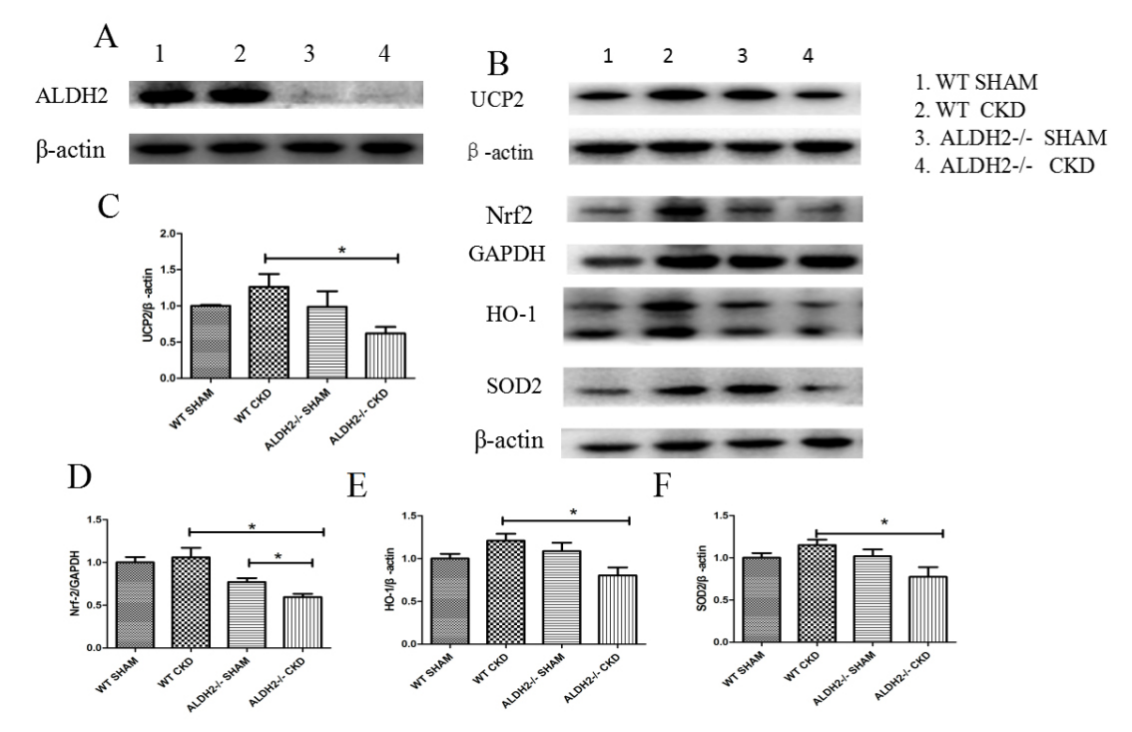
**Western blot analysis of ROS related proteins expression of WT and ALDH2/- groups post 5/6 NX**. (A) Representative blots of ALDH2 and β-actin (loading control). (B) Representative blots of UCP2, Nrf2, HO-1, SOD2 (loading control), B-actin (loading control), and GAPDH (loading control). Quantitative analysis of expressions of UCP2 (C), Nrf2 (D), HO-1 (E), and SOD2 (F). Values are presented as mean ± SEM (n = 3–6 per group). * *p *< 0.05. ROS, reactive oxygen species; WT, wild type; ALDH2, aldehyde dehydrogenase 2; GAPDH, glyceraldehyde 3-phosphate dehydrogenase.

Oxidative stress is an important risk factor contributing to myocardial and renal damages. The expression of ALDH2 was examined among these 4 groups. No difference was detected between the Sham and the 5/6 NX group both in respective WT and ALDH2-/- mice (Fig. 5A). By serving as critical endogenous antioxidant enzymes, Both UCP2 and Nrf2/ARE (antioxidant response element) could inhibit the generation of ROS and oxidative stress responses. As shown in Fig. 5B–F, the expression of UCP2 and Nrf2 were upregulated in the WT CKD mice, while loss of ALDH2 significantly reduced compensatory responses. Moreover, the downstream molecules of Nrf2, HO-1, and SOD2 were also increased in WT mice and decreased in ALDH2-/- mice at 12 weeks post 5/6 NX procedure. No statistically significant difference of Nrf2 was observed between WT Sham and ALDH2-/- Sham groups (*p* = 0.133). Collectively, our data provide evidences that ALDH2 deficiency could exacerbate CKD-induced cardiac dysfunction, possibly by inhibiting the UCP2/Nrf2 pathway.

## 4. Discussion 

The results of our study were as follows: (I) ALDH2 deficiency does not affect the process of compensatory cardiac hypertrophy induced by CKD, but resulted in cardiac dysfunction in this CKD model; (II) ALDH2 deficiency could exacerbate oxidative stress post CKD challenge, as evidenced by increased levels of ROS and decreased expression of antioxidants, including UCP2 and Nrf2, which might be responsible for the reduced cardiac function in this model.

Among the aging population, CKD is an increasing health problem, which in turn accelerates organ dysfunction among the elderly [22]. A previous study demonstrated that CKD was also linked with an increased risk of cardiac sudden death in elderly patients [23]. Clinical studies have also found that CKD patients usually manifest an increased left ventricular mass as compared with non-CKD patients [6]. The damages induced by CKD are commonly characterized by cardiac hypertrophy, arrhythmias, and heart failure [24, 25]. Among these, cardiac hypertrophy has been considered a compensatory response at the early stage [10], while contractile dysfunction and dilated left ventricular diameter often present after sustained CKD stress. In our study, only WT mice developed compensatory hypertrophic responses as evidenced by increased LVAWD and cross-sectional area (CSA). However, ALDH2-/- mice displayed decreased EF, as well as increased left ventricular diameters and fibrosis deposition with a similar left ventricular hypertrophy status to WT mice. Taken together, this model revealed that ALDH2 deficiency mice with CKD experienced heart failure without compensatory left ventricular hypertrophy.

**Fig. 6.**
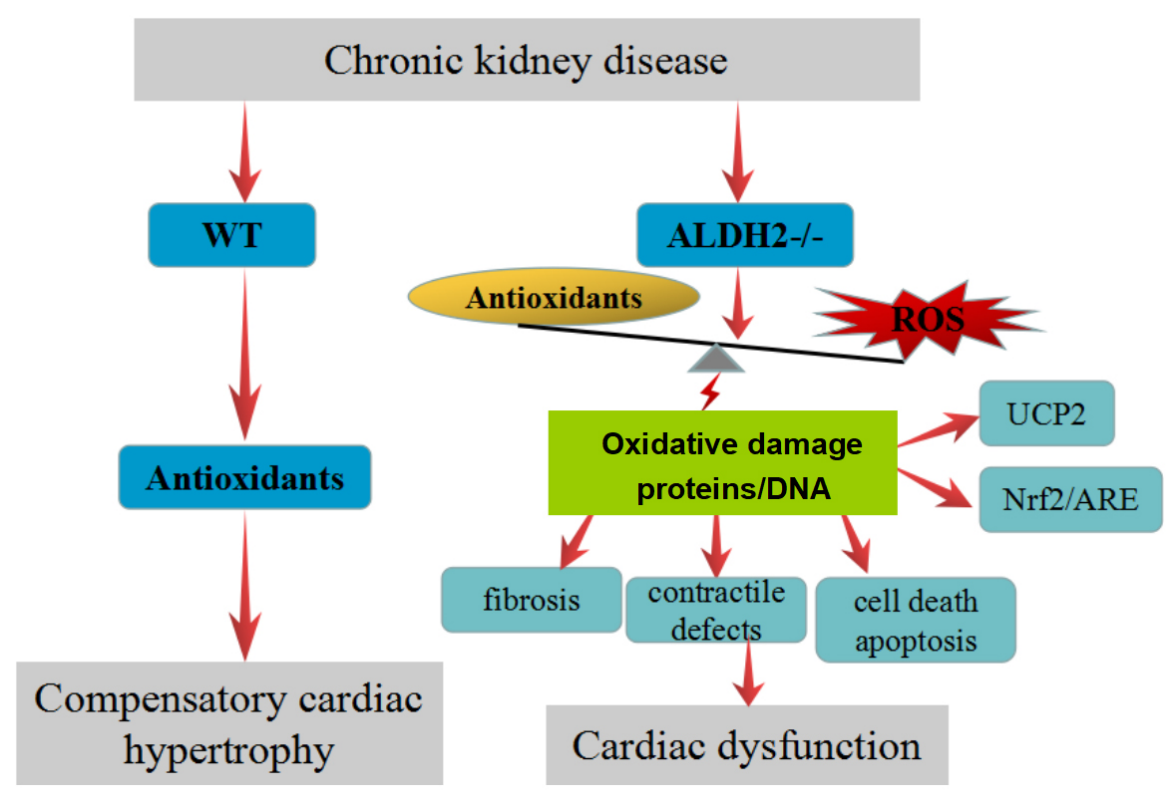
**Schematic diagram picturing the role of ALDH2 in CKD-induced cardiac dysfunction**. ALDH2 deficiency exacerbates CKD-induced cardiac dysfunction via down-regulate UCP2 and Nrf2/ARE induced ROS-dependent cardiomyocyte apoptosis.

The pathogenesis of cardiac dysfunction in CKD patients is complicated, involving chronic activation of the renin-angiotensin-aldosterone system (RAAS) and the sympathetic nervous system (SNS), elevation of nephrotoxic substance in serum, or reduced renal perfusion [24, 25]. Chronic activations of the RAAS and SNS have been well documented in the pathogenesis of heart failure caused by cardiomyocyte hypertrophy, inflammation, apoptosis, and fibrosis in the heart [26, 27]. Besides, RAAS and SNS have been shown to also capable of damaging the mitochondria and increasing oxidative stress [19]. Notably, increasing data have been reported that the cardiomyopathy associated with CKD is commonly associated with excess levels of ROS [28]. Our study findings are consistent with previous research, and showed that the oxidative stress post CKD exposure was further exacerbated in the situation of ALDH2 deficiency. The main source of ROS production is mitochondria, in which the mitochondrial uncoupling proteins (UCPs), locating in the inner mitochondrial membrane, have been demonstrated as essential regulators of ROS production [29], thus potentially responsible for the mitochondrial oxidative metabolism and uncoupled proteins in the failing heart [30, 31]. One of the most popular isoforms in UCP family, UCP2, was found in various tissues, including those of the central nervous system, macrophages, kidney, spleen, and thymus [32]. Although the physiological role of UCP2 has not yet been fully elucidated, accumulating evidences have shown that it could reduce mitochondrial ROS generation and cardiomyocyte apoptosis via stimulating proton leak and thereby ameliorating cardiac function. A member of transcription factor Cap ‘n’ Collar (CNC) family, Nrf2 controls the expressions of various anti-oxidative genes and enzymes, including SOD-2 and HO-1, and decreases the production of ROS [18, 33]. In our study, we observed an increased expression of UCP2 and Nrf2, as well as the antioxidants (SOD-2, HO-1) in the hearts of WT mice post CKD exposure, while ALDH2 deficiency significantly reduced the expressions of UCP2, Nrf2, SOD-2, and HO-1. Thus, our results suggested that increased oxidative stress by the ALDH2/UCP2/Nrf2 axis could exacerbate CKD-induced cardiac dysfunction in the case of ALDH2 deficiency (Fig. 6).

In addition, the role of diet, such as carotenoids supplementation, was also be considered to influence the oxidative stress for their free radicals scavenger properties and their skills in improving low-density lipoprotein cholesterol resistance to oxidation [34]. However, in our study all groups were fed with normal diet. In future, we can further investigate the relations in subgroups according to the diet difference.

There were some limitations to our study. Lack of serum and urine parameters (serum and urine creatinine and serum urea levels, urine protein level and creatinine clearance) make the severity evaluation of CKD difficult in these animals. All the echocardiography parameters looked similar between the WT CKD and ALDH2-/- Sham mice, and different from WT SHAM mice, which means that ALDH2 deficiency can affect the heart without any involvement of CKD. However, the comparisons between the WT Sham and ALDH2-/- Sham showed no statistically significant differences among the echocardiographic data and Nrf2 protein level. Increasing the number of animals in each group might alter the statistical significance between some groups. Moreover, as hypertrophy and dilation can represent stages of the continuum of events, it would be better to estimate cardiac function after a greater length of time had passed.

## 5. Conclusions 

In conclusion, our study revealed that ALDH2 deficiency increases oxidative stress and exacerbates CKD-induced cardiac dysfunction in mice post 12-weeks NX via downregulation of UCP2 and Nrf2/ARE signaling. People with ALDH2 inactive isoforms who are diagnosed with CKD should receive more intensive monitoring for potential cardiac damages. Some kind of strategy such as activating UCP2 and Nrf2/ARE pathways or anti-oxidative action maybe attenuate 5/6 nephrectomy induced cardiac dysfunction, even in the presence of ALDH2 deficiency.
